# A systematic review of the life cycle cost estimation of upgrading buildings for sustainability

**DOI:** 10.1007/s11356-025-36757-x

**Published:** 2025-08-11

**Authors:** Ali Tighnavard Balasbaneh, Willy Sher, Jingnan Li

**Affiliations:** 1 London South Bank University London, London, UK; 2 School of Architecture and Built Environment, College of Engineering, Science and Environment, The University of Newcastle (UON), University Drive, Callaghan, NSW 2308 Australia; 3 LSBU Business School, London South Bank University, 103 Borough Road, London, SE1 0AA UK

**Keywords:** Renovation, Retrofit, Refurbishment, Sustainability, Life cycle cost, Construction industry

## Abstract

In this study we have identified and evaluated literature related to refurbishment, renovation, retrofit and/or repair in the construction sector. We present a bibliometric evaluation along with a systematic critical review to identify materials used in upgrading buildings. We located 47 such publications. We found that more studies focus on renovation than those that address refurbishment, retrofit or repair, with respective counts of 19, 17, 12 and 10. The number of publications has fluctuated over the past few years. In some years, such as 2015, 2018 and 2023, an increasing number of studies were published, while there was a decline in 2015 to 2017 and 2020. Notwithstanding this, spikes in publication numbers occurred multiple times. Furthermore, the citation trend was generally upwards, suggesting that research in this field has gained increasing academic attention and influence over time. The originality of this study reveals the uncertainty of researchers in selecting cost evaluation methods when upgrading buildings. The trend suggests that further innovation and clarification is needed to enhance cost assessment methodologies. Evaluation of system boundaries revealed that most studies do not consider the end-of-life stage when evaluating different materials to upgrade buildings. To achieve an optimum building upgrade solution, single-objective optimization such as evaluating cost or LCA is insufficient, and multi-objective assessment methods need to be considered. Further research is needed to determine which upgrading strategies can effectively influence costs in optimization energy strategies.

## Introduction

Reducing the environmental impact from different industries has attracted considerable attention over the past few decades. Many adopted alternative protocols and strategies to control and mitigate climate changes (Singh et al. 2023) and move towards greener products and sustainability. Thus, sustainability assessment has become a favoured solution for evaluating emissions (Balasbaneh et al. 2022). The construction industry is an important contributor to climate change, as it consumes raw materials and produces waste (Lovrenčić Butković et al. 2021). Many previous studies have investigated sustainability in the construction industry. Some researchers have proposed alternative construction techniques in the UK (Hamilton-MacLaren et al. 2013) or replacing conventional materials with more sustainable ones (Balasbaneh et al. 2024). Despite numerous attempts to improve the sustainability of the construction industry, an optimal level is still elusive. The main concern hindering the adoption of sustainable practices is operating costs, which need to be consider when making design decisions (Hamilton-MacLaren et al. 2013). Sustainable strategies are unlikely to become popular in the construction industry if they attract higher cost than conventional methods or strategies (Häkkinen & Belloni 2011). Financial concerns are one of the main barriers impeding moves towards sustainable cities and construction. The upgrading and renovating of buildings is considered as one of the most impactful economical approaches worldwide (González et al. 2021). In this study, we focus on literature that addresses the financial issues and sustainability of upgrading buildings.

Oduyemi et al. (2018) evaluated costs in the construction industry, revealing that these are not limited to the design stage and that other stages such as renovation and replacement are the most important cost centres as well. Money is a sensitive issue in the construction industry, since the expenditure and distinct nature of each construction project is complicated (Gholizadeh et al. 2024). One form of attracting finance for projects is through private finance initiatives (PFI). PFI projects should integrate the principles of sustainability in every stage of development (Salleh et al. 2018). However, implementing PFI in the construction industry is contentious (Talib et al. 2018) mainly because of a lack of sustainability clauses in contracts. There is a strong association between sustainability and cost. There is a belief that green buildings increase the capital cost of projects. Thus, the overriding perception of sustainability in construction is that it is an expensive option (Dams et al. 2023). In rapidly developing countries, sustainable solutions are usually set aside due to unidentified economic benefits (Talib et al. 2018). The construction industry lacks a comprehensive model of sustainable renovation or maintenance (Macek, D 2010).

Growing urbanization has attracted worldwide attention because of the finite nature of resources (Hong et al. 2018). The costs of operating buildings are another longstanding concern in many countries. Furthermore, sustainable or green housing is rare and unaffordable for some societies (World Economic Forum (WEF), 2019). Okoro et al. (2024) believed that constructing sustainable and affordable housing is complicated by financial issues. Most recent studies show that optimization of building space and of interior design are important factors in renovating buildings (Jung et al. 2024).

For the purposes of this paper, it is necessary to address the conceptual differences between renovation, refurbishment, retrofit and repair. Renovation refers to restoring or modernizing an existing building without fundamentally altering its original purpose (EN-15643–1: 2021; Gonzalez et al. 2021; Kovacic et al. 2015). This may be to improve the aesthetic or functional aspects of a building while keeping its core structural elements unchanged. Renovation often focuses on adapting old or obsolete buildings to match contemporary lifestyles or standards (Gonzalez et al. 2021). Refurbishment aims to restore a building to good condition after a usage period, to maintain its value and to extend its longevity (Ostermeyer et al. 2013). Refurbishment concerns the upkeep and renewal of a building’s existing components. Retrofit differs from renovation and refurbishment by adding new technologies or systems to improve performance (Fregonara et al. 2016). Furthermore, retrofit ensures that buildings constructed in the past are able to meet current standards. It is a forward-looking approach that recognizes the limitations of earlier construction approaches and actively seeks to integrate them with current technological advancements (Hu 2023; ISO 21929–1: 2011). Lastly, repair is narrowest in scope as it primarily emphasizes fixing damage or restoring functionality for the affected parts of a building (Chiang et al. 2015; Wittocx et al. 2022). Repair is reactive, addressing immediate issues such as damaged or malfunctioning components. It ensures that the original condition of a building or component is maintained rather than improving or altering them in any significant way (Wittocx et al. 2022).

These four concepts have implications for both sustainability and financing. Specifically, renovation and retrofit are more aligned with adaptation or innovation to changing societal needs, especially regarding sustainability (Mjornell et al. 2014; Vainio and Nippala 2023). Retrofit could help address broader environmental concerns by improving a building’s energy efficiency and reducing its carbon footprint with new advanced technologies (Hu 2023). Caskey et al. (2016) found that the annual energy associated with heating was decreased by approximately 50% in a retrofitted single-family detached house. Moreover, renovations could be implemented through various green practices, such as using recycled or eco-friendly materials (Mjornell et al. 2014). In line with this, Amoruso and Schuetze (2022) argued that renovation must be attractive, profitable and aligned with sustainability criteria. They argued that renovating buildings to create extra usable space generated positive net present value, offering an eco-friendly and cost-effective alternatives to new construction.

Both refurbishment and repair could help extend the life of a building and reduce the need for new construction, thus conserving resources and minimizing waste (Masseck et al. 2024). Refurbishment of buildings is often seen as a major contributor to reducing negative environmental impacts while being economically promising (Ostermeyer et al. 2013). In particular, Loh et al. (2019) argue that enhancing the energy efficiency of buildings during refurbishment reduces carbon emissions more effectively than doing so in new buildings. As for repair, climate change tends to impact considerably on the construction sector, particularly regarding building repair (Barrelas et al. 2021). Extensive repairs resulting from climate change factors, such as the rise in global temperatures and extreme weather patterns, could be both costly and environmentally damaging (Hauashdh et al. 2024). In this regard, Balasbaneh et al. (2019) have focused on the feasibility of repairs to evaluate building costs in relation to environmental emissions after repairs in a flood-affected area. Through greenhouse gas analysis, they concluded that, in the event of a flood, precast concrete framing performs well by releasing less CO_2_ after the repair stage.

Despite numerous strategies and practices being established to address climate change and promote sustainability in the construction industry (e.g. Hossain et al. 2020; Jones et al. 2019; Vainio and Nippala 2023), such as carbon offsets and energy efficiency, the financial implications and cost-effectiveness of these strategies remain underexplored. These challenges underscore the necessity for a more comprehensive integration of financial analysis within sustainability initiatives so that economic aspects are prioritized alongside environmental goals (Fregonara et al. 2018). Despite the many roles established to control climate change and lead to sustainability (such as carbon offsets), cost of those strategies have not been the focus of attention. Improving the technical performance of buildings within financial constraints is one of the biggest challenges facing the building sector (Munguba et al. 2024).

In this study, we have focused on the ways in which the costs of upgrading buildings have evolved. We have assessed alternative methods of evaluating construction costs including the strategies used for renovation, retrofit and refurbishment. Concurrently, key terms including optimization strategies, energy evaluation and circular economy are discussed to highlight significant areas. Finally, a schematic is provided to illustrate the scope of existing research to improve understanding of how future research could be expanded in the context of upgrading buildings. In this regard, the present study contributes to existing literature by critically reviewing key strategies of financial considerations. It offers insights into how sustainable practices can be made financially viable and economically efficient, making them attractive for adoption by industry.

## Method

This research has focused on peer-reviewed publications which comprised articles, conferences and book chapters. It did not cover grey literature or listing from databases other than Scopus. Scopus is recognised as the most reputable source of scientific documents in this domain and is widely used by researchers. Scopus has been selected because it offers broader coverage and database size. It also includes a wide range of sources such as book chapters and conference proceedings. It has a strong interdisciplinary focus (Chamorro et al. 2025). We used VOSviewer_1.6.20_exe (released on October 31, 2023) to generate the keywords, countries of publisher and origin for co-occurrence, co-citation, co-authorship and bibliographic coupling.

### Principles and procedures

Our preliminary search identified 1256 documents. These were reduced to a total of 709 documents, based on a broader scope for the search. This comprehensive approach significantly increased the likelihood of capturing all relevant studies in the literature. These 709 documents included book chapters, articles, conference papers, erratum, letters and notes in the press as well as final publications. The literature search was conducted in mid-2024, with the most recent Scopus query performed in July 2024. Multiple keyword combinations were employed to collect the most relevant studies. This ensured that coverage was complete, thereby facilitating comprehensive analysis for this systematic review. Table 1 shows the keywords searched for this study.

**Table 1:** Keywords—search query for upgrading buildings

No	Keyword queries	Number of articles
1	“Sustainable” AND “Construction” AND (“Life cycle cost” OR “LCC”)	709
2	“Sustainable” AND “Construction” AND “Building” AND (“Life cycle cost” OR “LCC”)	400
3	“Sustainable” AND “Construction” AND (“Life cycle cost” OR “LCC”) AND “Building” AND “Renovation” OR “Refurbishment” OR “Retrofit” OR “Repair”	48
4	“Sustainable” AND “Construction” AND “life cycle Cost” AND “Renovation” OR “Refurbishment” OR “Retrofit”	38
5	“Sustainable” AND “Construction” AND (“Life cycle cost” OR “LCC”) AND “Building” AND “Retrofit” OR “Repair”	21
6	“Sustainable” AND “Construction” AND “Building” AND (“Life cycle cost” OR “LCC”) AND “Renovation”	21
7	“Sustainable” AND “Construction” AND “Building” AND (“Life cycle cost” OR “LCC”) AND “Refurbishment”	18
8	“Sustainable” AND “Construction” AND “Building” AND (“Life cycle cost” OR “LCC”) AND “retrofit”	12
9	“Sustainable” AND “Construction” AND “Building” AND (“Life cycle cost” OR “LCC”) AND “Repair”	8
10	“Sustainable” AND “Construction” AND “Building” AND (“Life cycle cost” OR “LCC”) AND “Upgrade”	2

### Systematic literature review

**Fig. 1:**
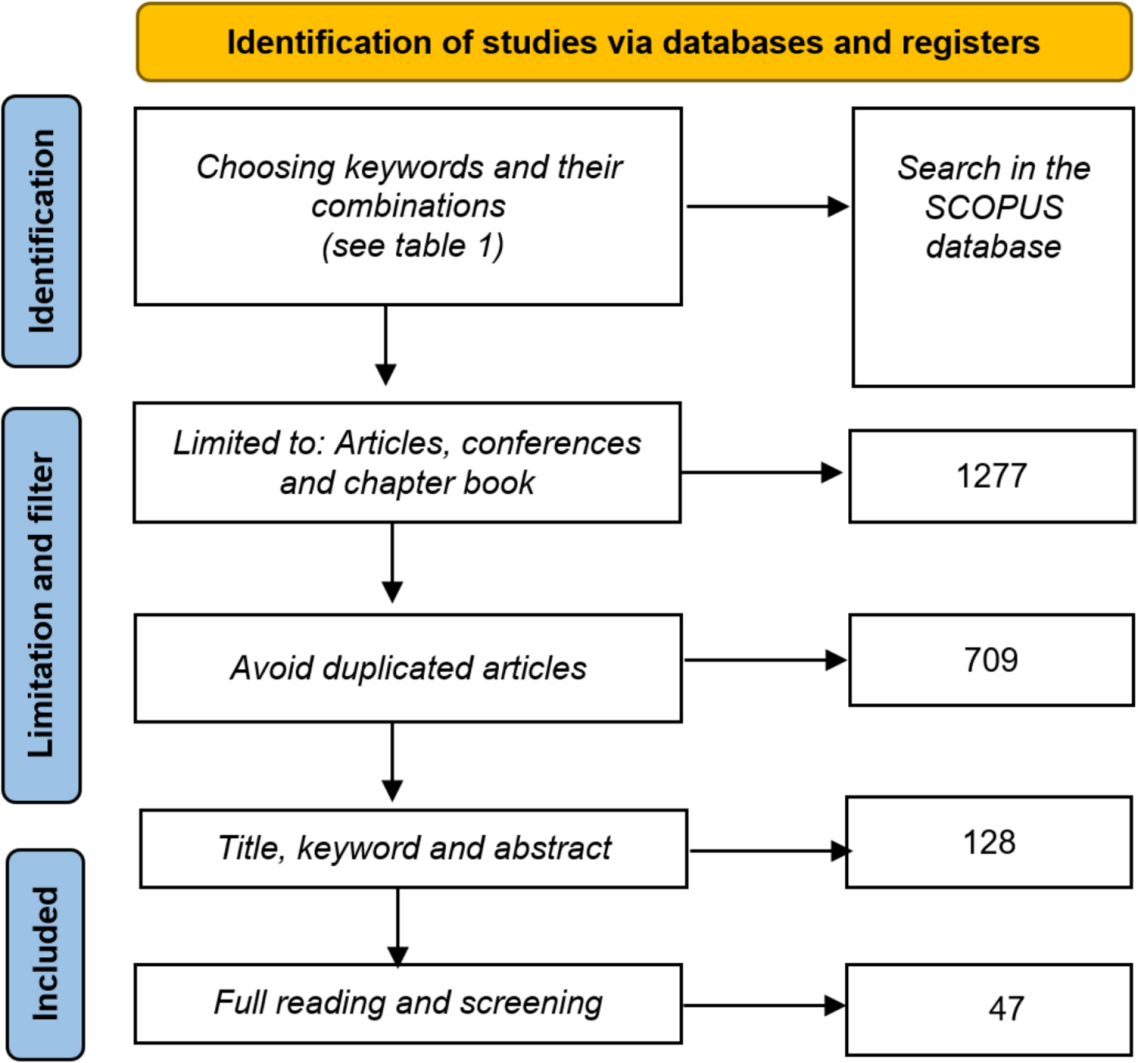
Screening process for selecting the relevant publications

Figure 1 shows the screening process used to locate relevant research articles. The search progressed through three stages. The first step after identifying the database to be used (Scopus) was to identify and extract relevant research articles (Singhania et al. 2023) via appropriate keywords and combinations thereof. These included relevant papers published between 2009 to mid-2024, in English and in peer-reviewed journals. Those excluded were studies published in languages other than English, unpublished papers, preprint publications and studies outside the research scope. In the “Limitation and Filter” stage, only academic articles, conference papers and book chapters were considered. Overall, 1256 articles are identified from 10 searches using the keywords shown in Table 1. Duplicate articles were then removed, reducing the number to 512 articles. In the final “Inclusion” stage, the articles were assessed based on their titles, abstracts and keywords. One hundred twenty-eight articles were retained. Reading the articles in full allowed us to filter out some irrelevant ones, leading to the final sample of 47 articles.

## Results

### Bibliometric data

**Fig. 2:**
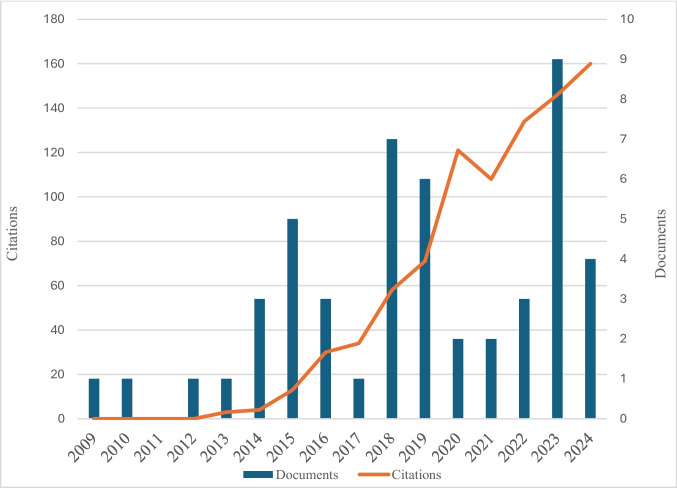
Data for number of publications and citation yearly

Figure 2 shows an up-and-down trajectory of publications on sustainability and cost in construction businesses between 2009 and July 2024. In particular, from 2009 to 2013, the number was relatively low and stable. In 2014, there was a noticeable spike of publications to 3. Subsequent spikes were in 2015, 2018 and 2023, respectively. The number of publications rises to 5, 7 and 9 in 2015, 2018 and 2023 respectively. Overall, the number of publications has fluctuated over the years, although the overall trend continues to increase. Moreover, Fig. 2 shows a steady increase in the volume of citations from 2009 to 2024. A particularly sharp rise in citations is observed in 2020, increasing from 71 to 121. However, during the same year, the number of publications decreased significantly, dropping from six in 2019 to only two in 2020. Moreover, the citation growth does not perfectly mirror the trend in publication numbers between 2015 and 2017, as well as between 2018 and 2022. Notably, the number of citations declined in 2021. Nevertheless, in 2023, there appears to be a clear correlation between the number of citations and publications. Overall, although spikes in publication numbers occurred multiple times, the citation trend was generally steady upwards, except for a temporary decline in 2021, suggesting that research in this field has gained increasing academic attention and influence over time.

In overall, as more publications have emerged over time, the topic has tended to become more central to both academic and industry discussions. The theories, models and empirical findings generated by previous studies are likely to be referenced by subsequent ones to build on and refine existing knowledge. Likewise, research on sustainability and cost has interdisciplinary relevance (Weaich et al. 2024; Reddy 2016). Such research is likely to be cited by studies from other disciplines, such as environmental science, engineering and business management, thus driving up citations beyond the core field. Firms operating in the construction industry have increasingly integrated sustainable practices into their core business activities, such as using green building materials, energy-efficient designs and waste reduction initiatives (Hossain et al. 2020; Jones et al. 2019). This might be because the construction industry has been criticized for causing adverse impacts on the environment (Lima et al. 2021), such as generating various kinds of pollution and waste (Cheriyan & Choi 2020; Wieser et al. 2021; Zuo et al. 2017).

To minimize negative environmental impacts, the construction industry has sought to introduce sustainable practices throughout its production chain and business operations (Lima et al. 2021). This trend potentially stimulates increasing attention from scholars across different disciplines to investigate the implications of sustainability in the construction industry (e.g. Barrelas et al. 2021; Balasbaneh et al. 2019; Hauashdh et al. 2024; Masseck et al. 2024). Considering the significant role of financing construction projects (Shan et al. 2017), it is not surprising to witness the growth of research aimed at understanding the cost and economic aspects of sustainable construction projects (Munguba et al. 2024).

From the perspective of academic scholars, the growing availability of data and advanced research methods could also enable scholars to publish in high-quality journals in recent years. Figure 2 highlights a significant increase in the number of publications in 2023. More sophisticated tools, such as AI-related data analytics, machine learning and big data, have become more accessible. These could potentially improve scholars’ ability to analyse large datasets and model more complex relationships for research topics. High-quality articles are more capable of extending the current understanding in a field and are likely to be cited and built on by subsequent studies. These studies are also likely to provide more contributions and insights, which could attract scholars’ attention and encourage potential future research directions. As a result, this could contribute to the increase in both the volume of publications and citations. Table 2 shows the main sources of research from 2009 to 2024 together with citations. The citations shown are those listed in Scopus and represent global citation counts.

**Table 2:** Main sources of global research from 2009 to 2024 with citations

Source	Documents	Citations	Total link atrength
Journal of Cleaner Production	5	272	30
Construction and Building Materials	1	105	9
Journal of Building Engineering	3	79	29
International Journal of Life Cycle Assessment	1	77	2
Sustainability (Switzerland)	2	61	14
Journal of Urban Technology	1	39	5
Building and Environment	3	37	14
Resources, Conservation and Recycling Advances	1	31	5
Innovative Infrastructure Solutions	1	29	7
Energy Procedia	1	19	0
Computing In Civil and Building Engineering—Proceedings of The 2014 International Conference on Computing in Civil and Building Engineering	1	18	4
Energy and Buildings	1	17	12
Sustainable Cities and Society	1	16	17
Sustainable Production and Consumption	1	11	11
Valori E Valutazioni	1	10	18
Facilities	1	9	27
Sustainable Construction Technologies: Life Cycle Assessment	1	9	31

Another plausible reason for this growth might be that stakeholders have emphasized greater environmental, social and governance (ESG) criteria in recent years. In the construction industry, in particular, stakeholders like investors or financial institutions who fund construction projects and provide financial capital are likely to pressure firms to highlight stakeholder interests (Jones et al. 2019). For instance, Jones et al. (2019) identified a significant industry transition towards environmentally beneficial practices among firms in the US commercial building industry between 1980 and 2019. Such an industry transition involves the contributions of numerous stakeholders, such as government agencies and large corporations. This might also lead to more academic interest in examining how construction firms fulfil different stakeholder demands by integrating the concept of sustainability into business operations (Jones et al. 2019; Rousseau et al. 2019).

**Fig. 3:**
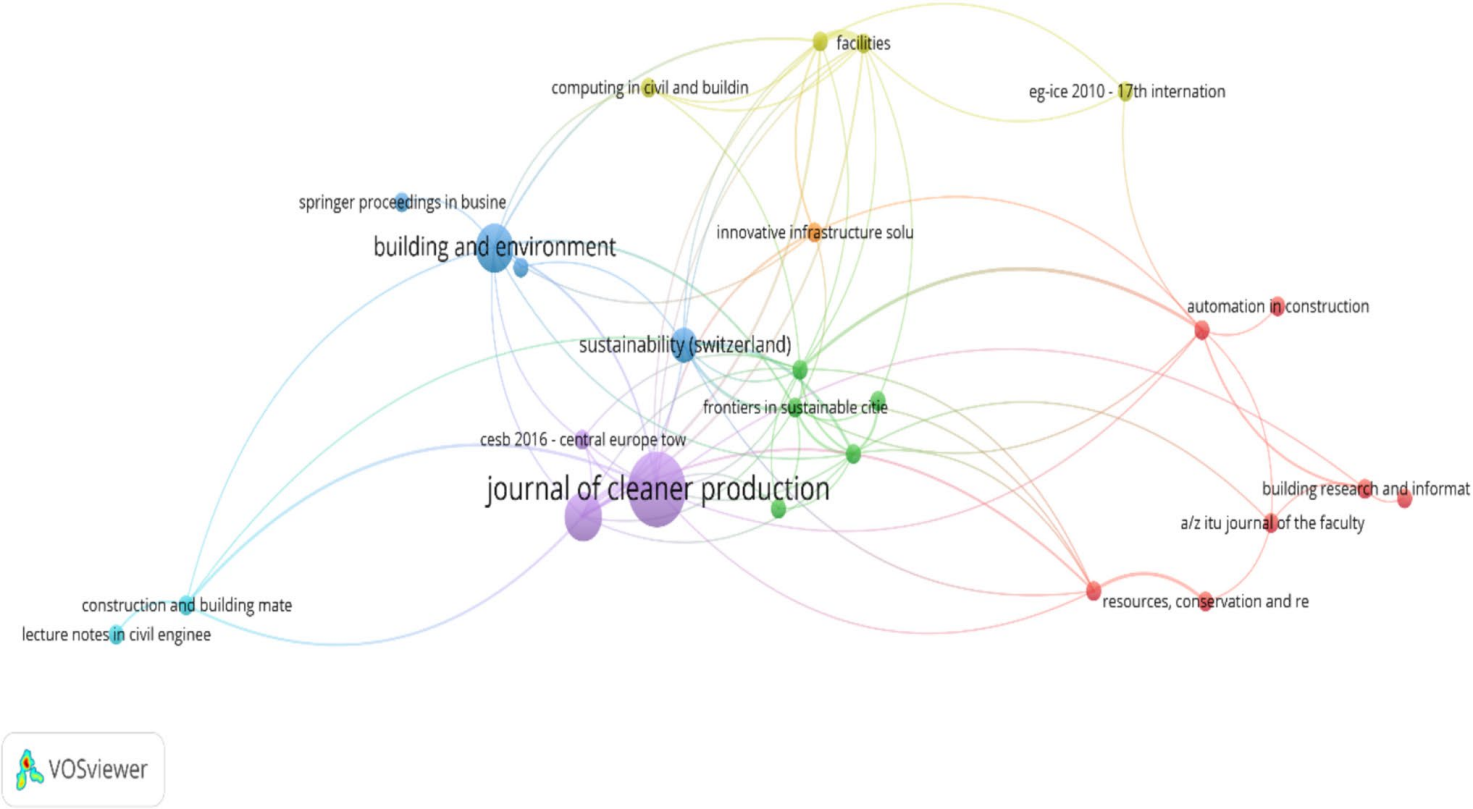
Bibliometric analysis network and link strength between publishers

Bibliometric analysis shows that the publications were spread across 40 journals in various disciplines, including sustainability, energy, engineering and technology. This further indicates that research on sustainability and financing in the construction industry has been multi-disciplinary. Scholars across fields have contributed to understanding this topic. Table 2 highlights the top 17 most productive journals based on the number of citations. From 2009 to 2024, the Journal of Cleaner Production published five articles that received 272 citations in total. This is followed by Construction and Building Materials and the Journal of Building Engineering, with 105 and 79 citations, respectively. The total link strength (TLS) measures how frequently a source is cited by authors in other articles. This indicates the degree of interconnectedness or influence within research network. In particular, the Journal of Cleaner Production, with a TLS of 30, appears to be a central hub for research on this topic. Such strong connectivity implies that the journal frequently engages with a broad spectrum of themes and bridges different disciplines. Also, this journal is likely viewed as a main source in sustainability-related discussions. Figure 3 shows the bibliometric analysis network and link strength between published articles. The total link strength metric represents the most significant relationships between publishers and their influential networks. The analysis of link strength reveals that the book *Sustainable Construction Technologies: Life Cycle Assessment* has the highest impact. This is followed by two journals, namely the *Journal of Cleaner Production* and the *Journal of Building Engineering*, respectively.

### Geographical distribution

The articles that met the selection criteria were from 38 countries. These were then ranked based on the citations they received. This indicates that the emergence and growth of the topic has been at a global level. Table 3 lists the top 14 countries contributing 43 of the publications. Most of the contributing countries are in Europe and North America. The USA and Italy had the most published articles with seven from each country. Articles from Sweden attracted the highest number of citations (139), followed by Belgium (114), Italy (84), Portugal (82) and Germany (77). Most attention has been historically paid to understanding the topics of sustainability and cost among developed Western countries. Developed countries typically have advanced economies and high literacy levels, which generally promote research on sustainability (Gao et al. 2021). It was therefore not surprising that countries like the USA would have produced extensive academic work in this field.

**Table 3:** Publications related to individual countries

Country	Documents	Citations	Total link strength
Sweden	3	139	41
Belgium	2	114	8
Italy	7	84	51
Portugal	2	82	16
Germany	2	77	34
Austria	3	42	3
United States	7	36	187
Spain	2	32	4
Saudi Arabia	3	30	161
Australia	2	18	94
United Kingdom	3	15	128
Czech Republic	2	12	4
Turkey	2	10	117
Canada	3	3	0

**Fig. 4:**
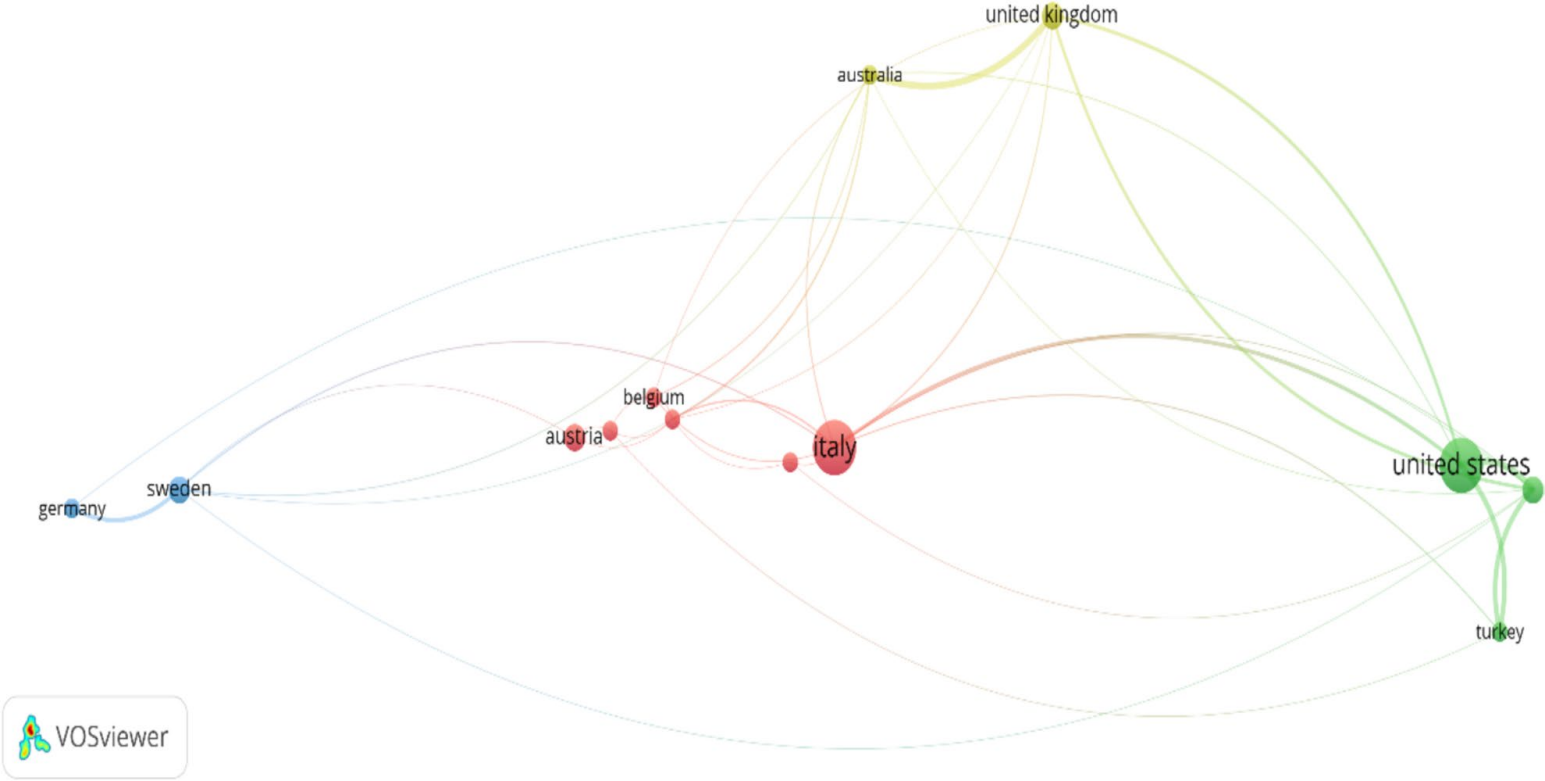
Bibliometric analysis network and link strength between countries

Moreover, the co-authorship between countries is illustrated in Fig. 4. There was close cooperation between some groups. Italy, Belgium and Austria formed a close cluster, representing a transnational pattern of authorship among them. The USA and Turkey were the second-largest cooperation cluster. Such collaboration helps promote a deep understanding of different topics because scholarly collaboration is the most formal way of intellectual association in scientific research.

According to previous study, collaboration among scholars is important to explore a field, and thus more cross-country collaborations are needed. Especially, as shown in Fig. 4, global collaboration networks allow developing countries to engage in the knowledge-building process that is traditionally initiated by developed countries (Palacios-Callender & Roberts 2018). This also benefits the quality of a study because of the joint efforts of multiple authors. An analysis of total link strength reveals that the USA has the highest value at 187, followed by Saudi Arabia with 161 and the UK with 128. This indicates that these countries have made the most significant contributions involving co-authors from various nations, demonstrating that the publications are not solely produced by individual countries.

### Co-occurrence

Each keyword was analysed using the software in which the occurrences and the total link strength were computed. The total link strength corresponds to the total number of references cited between one article and the others (Guo et al. 2019). These occurrences represent the number of articles in which a keyword was found. In fact, occurrence refers to the number of times each keyword is used in the target publications. For example, keyword “Life Cycle Costing” appears in the metadata (title, abstract or keywords) of 46 documents, and its occurrence is 46. Table 4 shows the keywords with the highest number of occurrences. Specifically, life cycle assessment, life cycle costing, sustainable development, costs, energy efficiency and sustainability were among the most highly co-occurring keywords with occurrence weights (total link strength) of 67 (730), 46 (421), 29 (312), 24 (254), 12 (146) and 12 (129), respectively. These most frequently co-occurring keywords indicate that the topics are closely interrelated in sustainability research. Previous studies have emphasized the cost aspects of sustainable development. This also highlights the popularity of integrating sustainability into economic planning and industrial practices (Stanitsas et al. 2021).

**Table 4:** Nominated keywords and occurrences

Keywords	Occurrences	Total link strength
Costs	24	254
Energy Efficiency	12	146
Sustainability	12	129
Sustainable Development	29	312
Repair	5	41
Retrofitting	5	59
Zero Energy Buildings	3	34
Intelligent Buildings	9	107
Investments	7	99
Decision Making	9	102
Life Cycle Assessment	67	730
Life Cycle Costing	46	421
Maintenance	4	41
Greenhouse Gases	7	108
Refurbishment	4	54
Cost Benefit Analysis	9	96
Cost Effectiveness	5	77
Environmental Economics	4	52
Environmental Impact	8	116

**Fig. 5:**
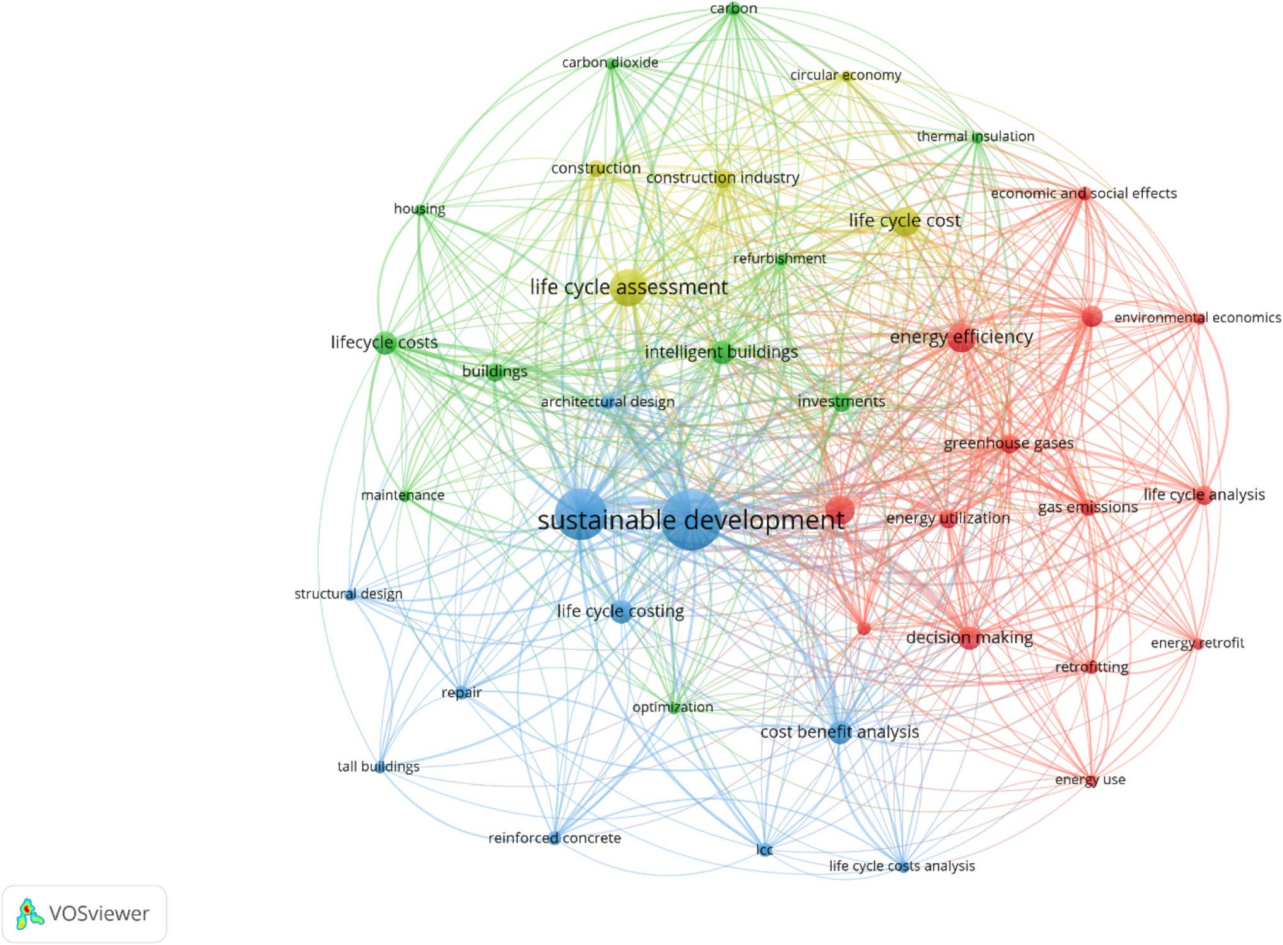
Co-occurrence map of the keywords

The co-occurrences represent the number of documents in which two specific keywords appear together. They measure the frequency of joint instances and are used to identify relationships and patterns between concepts. The co-occurrences of keywords may be visualised as a network of clusters, as shown in Fig. 5. All the keywords form four major clusters: cluster 1 (red), cluster 2 (blue), cluster 3 (yellow) and cluster 4 (green). The prominence of the circles and text in each cluster represents the strength of the co-occurrence with other keywords. While the distance between the items shows their relatedness, the lines represent the linkages between keywords. The keywords in each cluster were examined to establish the thematic distinguishable topic of the underlying keywords.

Cluster 1 relates to the economic and social aspects of energy use and decision-making. Energy efficiency, decision-making and life cycle analysis are the keywords with co-occurrences of 12, 9 and 7, respectively. A co-occurrence of 12 means that the keyword “energy efficiency” appeared in 12 of all the total articles. Studies on energy efficiency of different construction approaches emphasize the economic and social impacts of reducing energy use (e.g. Mangan & Oral 2014; Tagliabue et al. 2018). Improving energy efficiency lowers costs, reduces environmental strain and promotes social welfare by making energy more affordable and accessible. Importantly, energy saving is crucial for existing buildings, as these present huge potential for improvement through effective energy retrofitting (Tagliabue et al. 2018). Also, environmental economics highlights how economic incentives, policies and market mechanisms can be used to address environmental problems like pollution, resource depletion and climate change (Panza Uguzzoni et al. 2023). The goal is to understand the economic costs and benefits when promoting sustainable construction. For example, from an economic viewpoint, positive impacts are expected when self-healing concrete is used in building construction due to reduced maintenance and investments for repairing and replacement (Panza Uguzzoni et al. 2023).

Further, studies often conduct life cycle analysis to evaluate the environmental and economic impacts of a construction over the entire life span of buildings (Younis et al. 2018). The life cycle of a product begins with the production of raw material and extends to manufacture, use, transportation and waste management (Hauschild et al. 2018). Specifically, life cycle cost analysis is an established tool for minimizing the costs associated with the generation of a specific product (Fregonara et al. 2018). For example, Fregonara et al. (2018) suggest applying life cycle cost analysis to assess the economic sustainability of various technological construction scenarios. Moreover, Younis et al. (2018) conducted a life cycle cost analysis to compare the cost savings of structural concrete with seawater and recycled concrete aggregate in high-rise buildings, with traditional concrete mixes and reinforcement materials. In line with this, retrofitting, which includes upgrading existing systems to improve energy performance, is a practical application of life cycle cost analysis (Jafari et al. 2014). Jafari et al. (2014) examine how optimum selections of retrofitting activities could minimize the life cycle cost of a project. The decision to retrofit requires balancing economic costs like the investment needed with long-term savings, environmental benefits and social outcomes.

In cluster 2, the keywords with high co-occurrence weights are sustainable development (29), life cycle costing (46), costs (24) and so on. Some studies examine these terms by connecting them to architectural design and repair (e.g. Chiang et al. 2015; Wittocx et al. 2022). Sustainable development in architecture involves designing structures that minimize their environmental impact by using resources such as energy, water and materials efficiently, while promoting the well-being of occupants (Rousseau et al. 2019). Construction businesses aim to create adaptable and resilient buildings, ensuring they meet today’s needs without depleting resources for future generations. The goal is to promote long-lasting, environmentally responsible and economically efficient buildings. Also, a consideration of life cycle costing is essential when making sustainable architectural decisions (Younis et al. 2018). Construction businesses may assess the financial impact of their choices, such as selecting materials that have higher upfront costs but require less frequent repair or maintenance or using energy-efficient systems that reduce operational costs over time (Munguba et al. 2024).

Next, in cluster 3, topics primarily relate to life cycle assessment and the circular economy in construction businesses. Some frequently occurring keywords are life cycle assessment (67), construction (6), circular economy (4), refurbishment (4) and so on. Life cycle assessment (LCA) evaluates the environmental impact of buildings throughout their entire life span (Kmekova & Krajcik 2015). It often helps construction businesses make informed decisions by considering factors like energy consumption, material use and waste generation. This is particularly important in reducing the ecological footprint of construction projects, ensuring that environmental considerations are integrated into every phase, from design to demolition (Amoruso & Schuetze 2022). The prominence of LCA in this cluster reflects the growing emphasis on sustainability in construction practices.

In addition to LCA, the concept of the circular economy has gained increasing attention from scholars. The circular economy focuses on minimizing waste by promoting the reuse, refurbishment and recycling of materials. This approach contrasts with the traditional linear economy model, where resources are often used once and discarded afterwards (Fregonara 2023). Specifically, refurbishment highlights the industry’s shift to upgrading existing structures rather than demolishing them. It not only reduces material waste but also conserves energy. By adopting these sustainable practices, construction businesses aim to extend the life cycle of buildings while reducing costs and environmental damage, aligning with broader sustainability goals.

Finally, in cluster 4, some notable frequently occurring keywords include maintenance (4), housing (4), optimization (4) and carbon dioxide (4). Maintenance is crucial in ensuring that buildings operate efficiently over time. It promotes regular upkeep to extend the lifespan of a building and enhances its performance (Macek & Dobias 2014). Effective maintenance practices reduce overall energy and resource consumption and, consequently, lower carbon dioxide emissions (Chiang et al. 2015). Well-maintained housing could, therefore, become more sustainable, improving overall living conditions while minimizing environmental impacts. Optimization is another key focus in this cluster. It involves enhancing building performance through various strategies, such as installing energy management systems and technologies (Alothaimeen et al. 2023; Ostermeyer et al. 2013). Optimization can help decrease carbon dioxide output by improving energy efficiency during house usage. The interplay of these elements highlights the importance of integrating maintenance and optimization into different housing aspects to achieve sustainability goals and reduce carbon footprints effectively.

## Critical review

### Alternative economic indicators

**Fig. 6:**
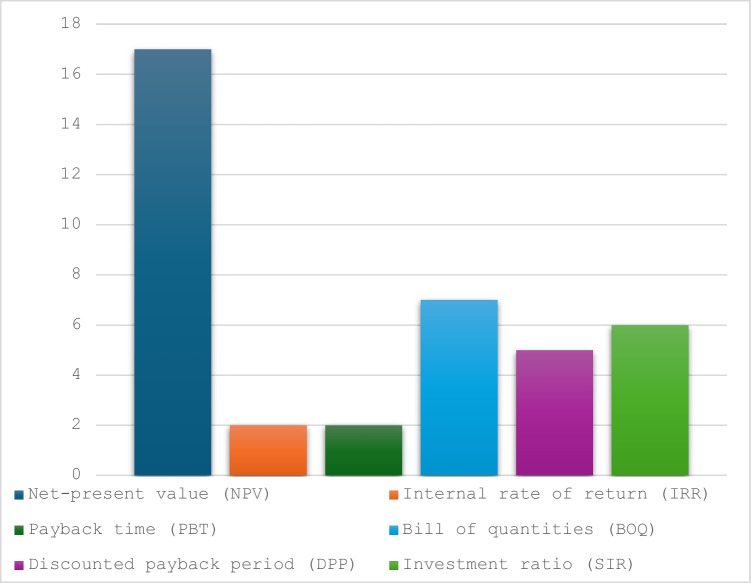
Alternative life cycle cost methods

Figure 6 shows the most popular methods of evaluating life cycle cost. Many different LCC methodologies may be used to assess alternative ways of upgrading buildings. These include net-present value (*NPV*), internal rate of return (*IRR*), payback time (*PBT*), bill of quantities (*BOQ*), discounted with inflation (*DPP*) and investment ratio (*SIR*). Net present value (*NPV*) is the most frequently used LCC indicator in the reviewed research papers. Other indicators such as savings to investment ratio (*SIR*) and payback time are also found in the literature.

Some studies did not indicate any specific method but merely mentioned using LCC analysis (Younis et al. 2018; Huang et al. 2018). For example, Chiang et al. (2015) conducted LCC of maintenance of sustainable building by calculating its initial cost and the present value of the replacement cost. The authors of another study determined cost using LCC to determine the total cost of ownership (*TCO*) (Kovacic et al. 2015). Additional studies used the discount rate (*DR*) as an indicator when conducting LCC (Ostermeyer et al. 2013). Despite most LCC analyses using *NPV* for assessing the cost of project, some studies argued that this method could not be applied when comparing different service lifespans (Schade 2003; Raposo et al. 2019). The authors of several studies believed that *NPV* is essential for LCC analysis to evaluate the total cost of owning, operating and maintaining a project or asset over its entire life span. It is clear that there is a need for a standardized reporting framework to identify which method is suitable for different upgrading approaches.

Regardless of the above, it is important to differentiate between the alternative methods shown in Fig. 6. The various methods of estimating costs such as *NPV*, *IRR*, *PBT*, *BOQ*, Period *DPP* and *SIR* can lead to different results because each method evaluates a project or investment from a different perspective and focuses on different factors. These methods measure different aspects of investments or project decisions. For example, while *NPV* and *IRR* focus on profitability, *PBT* focuses on liquidity, and *BOQ* on cost control. Therefore, using more than one method can provide a more comprehensive evaluation (Sokolov 2024). Thus, a sensitivity analysis should be conducted along with the cost estimation of building. Failing to do this will compromise the conclusion for the nominated upgrade strategy.

### Analysis of upgrade scenarios

The studies that considered the LCC of upgraded buildings also considered the LCA evaluation. Thus, the majority of studies applied LCA and LCC simultaneously. This reflects the sustainability philosophy that cost and environmental impact are two of three main pillars for sustainability evaluation. Table 5 shows publications on the life cycle cost and sustainability of renovation. Many articles emphasised the tremendous impact of existing buildings in Europe on economic and sustainability (Kovacic et al. 2015). Upgrading these existing buildings by renovating them is one of the possible and vital solutions towards more efficiency from the building sector. Renovation measures are often considered in light of repaying investments in a shorter time rather than taking into account life cycle costs. This is despite the fact that a thoughtful, comprehensive renovation is often more cost-effective in the long run (Mjörnell et al. 2014). Some renovations concentrate on reducing the energy consumption of buildings by providing either new solo technologies or combinations of strategies. For example, Mjörnell et al. (2014) applied ten different renovation strategies to decrease the energy consumption of residential multi-family buildings. These included simultaneously changing windows, ventilation equipment, advancing heating system and adding extra insulation. This resulted in renovations that marginally increased the costs of projects. Huang et al. (2018) assessed two strategies for renovating buildings namely, implementing low energy technologies and using of low environmental impact building materials such as structural timber.

**Table 5:** Publications on the life cycle cost and upgrade scenarios of renovation

Source	Method	Financial performance indicators	Analysis	Finding
Mjörnell et al. (2014)	LCA, LCC and social-LCA	*NPV*	Different renovation methods based on sustainability criteria	Renovation increased costs but lead to significant environmental and social benefits
Huang et al. (2018)	LCC and LCA	-	Applying low energy consumption	The operation stage has highest cost and environmental impacts
Amoruso and Schuetze (2022)	LCC and LCA	*NPV*	Three hybrid-timber building renovation	The renovations systems achieve negative global warming potential
Pernetti et al. (2021)	LCC	*NPV*	Near zero-energy buildings	Interest rate, maintenance costs and electricity prices have a largest impact on cost
Wouterszoon Jansen et al. (2022)	LCC and LCA	*NPV*	Building facade	Substituting materials for biological materials
Macek (2010)	LCC	-	Optimisation of renewal costs	Establish mathematical correlations for the minimization of costs
Alothaimeen et al. (2023)	LCC	Present value	Find the optimal solution considering costs	A proposed method can lead to optimal solutions
Oduyemi et al. (2018)	LCC	*NPV*	Level of awareness of LCC	LCC is a recognisable value for assessing initial costs, operating and maintenance
Gluch and Gustafsson (2015)	LCC	-	Evaluation of cost analysis in renovation stage	LCC is a suitable tool for conducting investigations
Aguacil Moreno and Rey (2020)	LCC	*NPV*, *IRR* and *PBT*	Renovation with photovoltaics	Renovation should be applied using renewable energy integration
Dragonetti et al. (2024)	LCC and LCA	-	Energy renovation in buildings	Energy will be saved after renovation
Jung et al. (2024)	LCC	-	User preferences in renovation	Construction cost account for 32.9%
Bartels et al. (2023)	LCC and LCA	-	Layered structures	Renovation of buildings and costs are dependent on DE-constructability
Vainio and Nippala (2023)	LCC, LCA	-	Best solution for renovation	Construction costs are a crucial factor in renovation
Kmet’kovä and Krajčik (2015)	LCC	-	Alternative thickness of facade and roof	Increase the overall quality of housing
Alshamrani and Alshibani (2020)	LCC and LCA	*NPV*	Renovation and major repair costs	Returned salvage value

Timber and engineering wood has been proven to be advantageous, as it decreases environment impact (Tighnavard Balasbaneh and Ramadan 2024). Building components such as cement, steel and windows contribute the least to building costs, compared to the operation stage. However, these components can profoundly decrease the environmental impacts. González et al. (2021) proposed renovation by installing external thermal insulation combined with rooftop solar photovoltaic (PV) systems, resulting in high payback periods in excess of 20 years. A key omission when calculating costs is that of the salvage value of materials (Alshamrani & Alshibani 2020). Their salvage value has been omitted from the majority of the articles included in this review and should be considered in future research.

Table 6 shows publications on life cycle cost and upgrade scenario of refurbishment. The majority of existing buildings in UK do not meet current sustainability requirements. The cost of refurbishment is the main obstacle impeding the upgrading of these buildings (Loh et al. 2019). Studies that focus on building materials such as brick or concrete could be useful for stakeholders in this regard (Balasbaneh et al. 2019). Some other studies have evaluated the costs of refurbishment. Buyle et al. (2019) assessed alternative wall assemblies considering refurbishment every 15 years. They found that metal sub-structures cost around 17% less than conventional walls. Other studies have defined a variety of upgrading approaches including using of refurbishment and retrofit simultaneously (Chiang et al. 2015; Ferreira et al. 2015).

**Table 6:** Publications on life cycle cost and upgrade scenario of refurbishment

Source	Method	Financial performance indicators	Analysis	Finding
Buyle et al. (2019)	LCC and LCA	Consequential	Alternative wall assemblies with substructure and finishing	Sand-lime bricks with metal studs have the lowest initial cost if refurbishment is consider for 15 years
Ostermeyer et al. (2013)	LCC and LCA	*DR*	Heating and ventilation	Social-LCA needs to support LCA and LCC
Chiang et al. (2015)	LCC and LCA	*DR*	Minimize costs	Propose a methodology to identify lowest cost and carbon emissions
Ferreira et al. (2015)	LCC and LCA	-	Assessing traditional refurbishment	Structural refurbishment seems to be environmentally more positive
Kovacic et al. (2015)	LCC and LCA	*TCO*	Alternative structural and thermal	Structural refurbishment has a positive cost impact
Balasbaneh et al. (2019)	LCC and LCA	*DR*	Alternative structural materials	Concrete costs less to repair than timber
González et al. (2021)	LCC	*NPV* and *BOQ*	Facade energy retrofitting	Payback period is lengthy
Raposo et al. (2019)	LCC and LCA	-	Seismic reinforcement in an existing building	Costs are reduced compared to the construction of a new building
Kim (2019)	LCC and LCA	-	Using BIM for simultaneous LCA and LCC for refurbishment	Recycled materials can be considered as refurbishment materials
Kim and Park (2018)	LCC and LCA	*NPV*	Using BIM for simultaneous LCA and LCC for refurbishment	BIM is a suitable platform to enable trade-off relationships between LCC and LCA simultaneously
Heidenthaler et al. (2019)	LCC	-	External thermal insulation composite system (ETICS) and radiators	The erection cost for building facades need to be reduced to below 36% to be considered as economically competitive
Masseck et al. (2024)	LCC and LCA	-	Sustainability of waste-based shading devices	Project contributes to defining more sustainable facades
Fregonara (2023)	LCC	*NPV*	Monetizing and modelling the discounted cash flow analysis (DCFA) of embodied energy	Economic factors must be considered in decision-making processes from early design to the end-of-life stage
Loh et al. (2019)	LCC	*NPV*	Develop an approach to select the most energy efficient material	50% saving in building life cycle energy cost over 40 years can be achieved
Halder et al. (2012)	LCC	-	Create a model for quick estimation of cost	LCC calculations give significant benefits in the early planning phases and help to reduce building operation and maintenance costs

Table 7 shows publications on life cycle cost and upgrade scenario of retrofit. The practice of converting existing housing to low energy may be called retrofit (Jafari et al. 2014). Life cycle cost assessment makes it possible to establish alternative costs of construction. When conducted during design it can impact on decisions to convert buildings to the green alternatives. Tagliabue et al. (2018) conducted a retrofit study to improve the building envelope using insulation replacement such as solar heat gain coefficient reduction, glazed surface replacement and thermal transmittance (*U* value) enhancement. They found that brick with raw earth has better performance than platform system wall.

**Table 7:** Publications on life cycle cost and upgrade scenario of retrofit

Source	Method	Financial performance indicators	Analysis	Finding
Fregonara et al. (2018)	LCC and LCA	*NPV* and *PBT*	District heating by gas cogeneration, photovoltaics and solar heating energy	Photovoltaic panels are the most sustainable energy-wise
Hu (2023)	LCC	-	Retrofit zero energy building	The operational life stage was a major contributor to energy consumption (82%) but not to the LCC (18%)
Iswidyantara and Husin (2023)	LCC	-	Green retrofit infrastructure	Factors that affect cost performance include: planning and energy
Fregonara et al. (2016)	LCC and LCA	-	Most preferable design for energy-efficiency	Proposed methodology for energy improvement in newly constructed or existing buildings
Jafari et al. (2014)	LCC	Monte-Carlo	Monte Carlo simulation for estimating LCC	Cost of buildings may increase if sustainable retrofit approach selected, but long-term costs would decrease
Tagliabue et al. (2018)	LCC and LCA	*NPV* and *DPP*	Wall envelope components	Brick with raw earth is 60% of platform system wall
Munguba et al. (2024)	LCC	*NPV* and *PBT*	Photovoltaic/near-zero energy	Minimize energy use intensity (EPI) achieved $412,978 net present value
Mangan and Koçlar Oral (2014)	LCC	*DPP*	Energy retrofit	Annual usage cost is reduced 23–26%
Younis et al. (2018)	LCC	*DR*	Cost of structural concrete using seawater	Results were found to be highly sensitive to the assumed discount rate and construction costs
Panza Uguzzoni et al. (2023)	LCC	*NPV*	??	??
Wittocx et al. (2022)	LCC and LCA	*NPV*	Renovation practice of existing concrete structures	Patch repair is the best option for concrete repair
Zhu and Feng (2023)	LCC and LCA	*NPV*	Integrated approach of using BIM with LCA and LCC is proposed	Building operating costs can be reduced

**Fig. 7:**
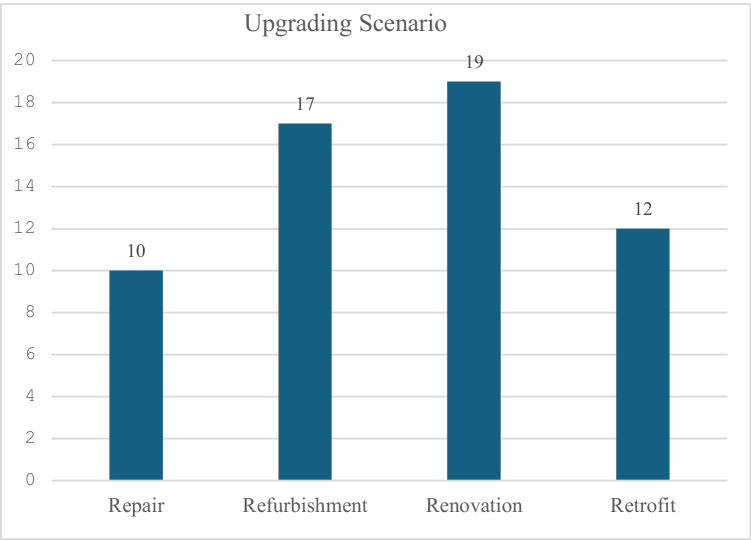
Different upgrading scenarios considered in review

Few studies of the maintenance and renovation of concrete structures were found to have been conducted (Wittocx et al. 2022). In addition, few studies of the cost of refurbishment, renovation and repair of other materials were found. Wittocx et al. (2022) conducted a LCC study of alternative repair techniques such as galvanic cathodic protection, conventional repair, patch repair and total replacement. They found patch repair to be the most economic option, where the lifetime of balconies was considered as 5 years. Zhu and Feng (2023) explored the trade-offs between energy savings, various environmental impacts and cost effectiveness of assessing building upgrades. They showed that energy-saving measures reduce building operating costs by about C$4000. Figure 7 shows that the articles on renovation outnumber those on refurbishment by 19 versus 17, respectively. The number of articles about retrofit and repair is 12 and 10, respectively. However, many studies use ‘renovation’ and ‘retrofit’ interchangeably (Kmet’kovä and Krajčik 2015).

### System boundary

Table 8 shows those studies that considered the system boundaries of building renovations based on the EN15978 standard. We analysed the system boundaries used in the LCC articles for this study. Some articles evaluated most building stages namely, construction, maintenance, operation and end-of-life activities (Huang et al. 2018). For example, Amoruso and Schuetze (2022) considered the construction cost (A1–A5) and demolition cost (C1–C4) for renovation of modular structures. Amoruso and Schuetze, (2022) assumed that the cost of demolition is 2.50% of capital expenditure of construction cost.

**Table 8:**
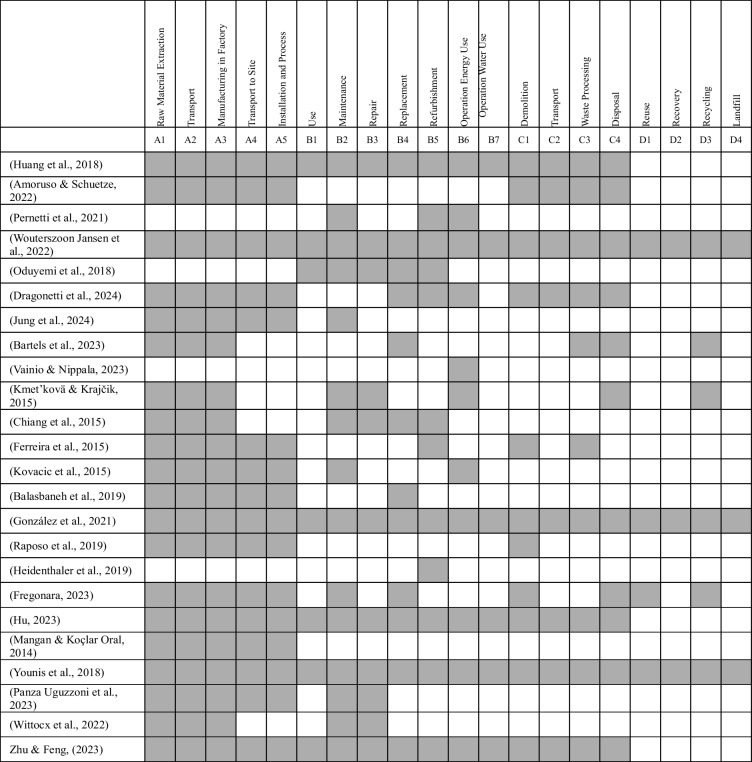
System boundaries based on the EN15978 standard

The authors of previous studies make many assumptions when evaluating the costs of different stages of a building’s lifespan. This highlights the importance of conducting a sensitivity analysis to verify results. For example, Pernetti et al. (2021) argued that in the absence of reliable data, maintenance costs could be applied based on standard values from literature, which corresponded to 1.5% of the construction cost. Most LCC studies were found to neglect the costs of planning buildings, which included the preparation of project budgets. This is also an important stage when estimating the costs of upgrading existing buildings. For example, Kmet’kovä and Krajčik (2015) considered the cost of planning for building and investment showing a high proportion of cost to be for renovation.

Bartels et al. (2023) observed that individual life cycle phases (such as design and construction) are often considered when assessing building cost. This is confirmed in this current study, as shown in Table 8. To enable stakeholders to make fully informed decisions, there is a need to consider the whole life cycle of buildings by considering different upgrading strategies. They can then choose the optimum solution when deciding between renovation or refurbishment of buildings.

In general, the salvage value of materials is rarely addressed in previous studies. Some simply assumed that waste disposal costs and salvage values would cancel each other out and did not make any allowance for salvage values (Koo et al. 2014). Furthermore, salvage value was not mentioned in any upgrading study, where it would seem to have a considerable impact on project costs. It is recommended that salvage value is taken into consideration (Alshamrani & Alshibani 2020). This would enable a more realistic cost to be calculated. A limited number of studies did consider the salvage value of materials and highlighted the importance of this cost element. Alshamrani and Alshibani (2020) considered the salvage value to be $0 for wood-system buildings, $11.4 million for conventional precast concrete system (CC) to $16.5 million for green precast concrete system (GCC), $12.3 million for sustainable steel systems (GSC) to $5.3 million and for conventional steel system (SS) after 20 years of operation for Saudi Arabia. Finally, the salvage value of sustainable materials needs to be compared to conventional materials when evaluating initial investments. An eventual gain of $5.1 million represents the future attainable return in the salvage of sustainable precast concrete system, compared to conventional precast concrete facility. This shows the importance of taking into consideration salvage value when evaluating the cost of projects. Meanwhile, future studies on photovoltaic panels, as noted by Niemczak et al. (2023), should assess more details of their costs and advantages for residents and consumers. These studies should also analyse the payback period of these investments under various climate conditions.

Conventional demolition of existing structures can lead to high costs for stakeholders and significantly increase waste generation. Therefore, studies that do not consider recycling, reuse or material recovery may result in higher final costs for building upgrades. This aspect could be reassessed through sensitivity analysis. In the context of a circular economy, materials can be reused, recycled or resold, which may generate financial benefits and reduce overall project budgets. In conclusion, the majority of articles reviewed did not consider recycling, reuse and disposal costs. This makes it very difficult to manage the recycling and waste management costs for entire buildings as well as for individual building materials. Furthermore, the conditions in every country are different making it difficult to compare the relevant waste cost data.

### Optimization strategies

Building is the most costly of all industries. It accounts for nearly 40% of dioxide carbon emissions (CO_2_eq), 70% land change and 50% of material flows (UNEP 2019). Therefore, any improvement in this sector will provide a valuable improvement to planet and human health and the economy. Some research has been conducted into optimization strategies or methodologies related to the economic cost of buildings. These studies have concentrated on alternative methodologies for optimizing project cost. They argue that single-objective optimization cannot address the needs of projects and should strongly be avoided. For example, Ostermeyer et al. (2013) conducted a life cycle sustainability assessment (LCSA) for refurbishments using an optimization method to combine LCA, LCC and social-LCA. Alothaimeen et al. (2023) applied an optimization tool to deliver an optimal solution for the LCC and sustainability of construction projects. Bartels et al. (2023) believed that BIM simulations of LCC and LCA are an efficient optimization method of estimating costs. Chiang et al. (2015) conducted a methodology to minimize project cost along with labour impact and carbon emission for building refurbishment and maintenance. Jung et al. (2024) considered that renovation should focus on optimizing building space, highlighting the importance of interior design and architectural planning. Munguba et al. (2024) claimed that using photovoltaics in building retrofits could lead to cost optimization. Tagliabue et al. (2018) believed that the heat storage capacity or thickness of wall insulation has the potential to optimize building cost. Wouterszoon Jansen et al. (2022) believed that optimizing the reuse of technical materials is an effect sustainability method. Zhu and Feng (2023) believed that heat pumps have a higher economic benefit, compared to adding more wall insulation.

### Energy evaluation

As the world’s population and energy consumption rise globally, upgrading existing buildings presents a promising pathway to sustainable practice (Munguba et al. 2024). However, balancing a building’s technical performance with financial constraints is very challenging. The energy performance of buildings is among the most important renovation topics (Dragonetti et al. 2024; Vainio and Nippala 2023). Table 9 provides an evaluation of energy interventions in upgrading buildings. The main purpose of considering optimizing energy during upgrades of buildings is to move towards sustainability. Dragonetti et al. (2024) analysed the LCC of renovation of a student house in Greece, showing that energy cost savings of up to 50% post-renovation. Mangan and Koçlar Oral (2014) evaluated the cost of the energy performance of buildings retrofitting with insulation for exterior walls, glazing systems, solar control and PV panels. They found that the operational cost had decreased by 23–26% on average. Mangan and Koçlar Oral (2014) claimed that the annual usage cost is reduced 8–12% after retrofitting. Munguba et al. (2024) used photovoltaic strategy for renovation to achieve near-zero energy buildings. This reduced energy consumption by more than 45 MWh/year, equivalent to $170,000 in net present value.

**Table 9:** Evaluation of energy in upgrading buildings

Source	Upgrade scenario	Finding
Dragonetti et al. (2024)	With and without interventions	50% cost reduction
Mangan & Koçlar Oral (2014)	Installing insulation for exterior walls, glazing systems, solar control and PV panels	A reduction of 28–30% in annual energy usage
Munguba et al. (2024)	Photovoltaic/near-zero energy	Energy and financial metrics can simultaneously maximize energy savings
Vainio and Nippala (2023)	Zero energy ground	Replacing existing buildings with new must be done only where dangerous situations threaten current ones
Mjörnell et al. (2014)	Heating systems, building envelopes, ventilation systems and radiators	Proposed a new tool for assesing the energy
Huang et al. (2018)	Increasing renewable energy supply and carbon taxes	Renovations can effectively impact costs
Hu (2023)	Net-zero building	Cost of operation stage is responsible for only 18% of total cost

Vainio and Nippala (2023) argued that the energy efficiency resulting from connecting a building to district heating justifies the structural improvements required. Mjörnell et al. (2014) applied three different renovation approaches. However, their results did not distinguish between the alternative approaches. Huang et al. (2018) suggested applying near zero-energy and carbon taxes as an economic incentive for buildings. Hu (2023) claimed that the operational life stage in net zero buildings is responsible for 82% of the whole building energy cycle. This is while the cost of this stage is only responsible for 18% of total cost. Renovation can therefore be a reasonable approach to improving existing buildings. An analysis indicated that during the whole building life span, the operational life stage (B6) was a major contributor to life cycle energy (LCE) by 82% and life cycle carbon emissions (LCCE) by 77% but not to life cycle cost (LCC) (18%).

One of the most interesting topics in building renovation is energy saving which is commonly considered as cost saving. For example, previous studies have claimed that energy saving strategies when retrofitting buildings leads to lower building operating cost (Teamah et al. 2022). In contrast, other researchers (Zhu & Feng 2023) have demonstrated that spending more on retrofitting or upgrading may not lead to lower energy costs. Kmet’kovä and Krajčik (2015) investigated energy saving for retrofitting buildings by various means method including changing the thickness of insulation for walls and roofs. Their calculations showed a reduction of more than 50% of energy costs. Tagliabue et al. (2018) demonstrated an energy saving of 19% with a combination of a ventilated slab floor, wooden roof and brick wall with raw earth. Thus, there is need for more studies to show which upgrading strategies can effectively impact costs.

### Social evaluation of upgrading buildings

The final pillar of sustainability, social impact, has received negligible attention (Mjörnell et al. 2014), as shown in Table 10. The initial purpose of social aspect is on trust, justice, well as fair living standards, health and civic participation (Chiang et al. 2015; Ostermeyer et al. 2013). Some argue that social impact could be considered in the renovation of buildings and that this could be of benefit when selecting desirable choices (Amoruso & Schuetze 2022). Most studies only suggest implementing social impact rather than its application.

**Table 10:** Social methods of upgrading buildings

Source	Scenario	Criteria
Mjörnell et al. (2014)	Renovation	Proposes SLCA as a framework
Kovacic et al. (2015)	Renovation	Accessibility, assisted living suitability, rent level affordability and social utility
González et al. (2021)	Refurbishment	Acoustic mitigation of construction works, impact of construction work towards neighbourhood, social fairness in construction stage and use of local workforce

Mjörnell et al. (2014) encouraged house owners to consider not only environmental and economic criteria but also to consider the social aspects of different renovation alternatives. Mjörnell et al. (2014) used the S2020 Matrix (Kunskapsmatris 2020) approach as a qualitative methodology to evaluate social impact. González et al. (2021) evaluated refurbishment of social assessment based on the MEFA (CEN 2014) approach and considered the following criteria: acoustic mitigation of construction works, impact of construction work on neighbourhoods, social fairness in construction and use of local workforces. Masseck et al. (2024) conducted social assessment of refurbishment by assigning points without relying on SLCA because it was not possible to use SLCA, as it lacked an appropriate and mature database. Kovacic et al. (2015) considered social sustainability by including criteria such as accessibility, assisted living suitability, rent level affordability and social utility.

### Circular economy analysis

An important criterion for sustainability is the circular economy. This has a significant impact on the costs of projects, especially in the refurbishment or renovation stage (De Souza Rocha et al. 2024; Kong et al. 2024). Circular economy principles have been included in some publications that consider costs. The Circular Economy Action Plan is a comprehensive strategy to promote more sustainability (Dragonetti et al. 2024). By renovating rather than demolishing, the lifespan of a building is extended, which aligns with the circular economy’s emphasis on prolonging the life of products and materials.

For example, Wouterszoon Jansen et al. (2022) evaluated a circular renovation using LCC to compare skin variants and kitchens, expressing their results as total costs (*TC*). They showed that the renovated facade with the lowest cost of manufacture was not necessarily the best cost option as the installation cost might be higher than for other facade systems. This shows the importance of considering all the lifecycle stages of components and materials to achieve the optimum cost benefit. Buyle et al. (2019) investigation of the circular economy assumed 5% material loss for every refurbishment with direct reuse. Transition to the circular economy could be achieved by valorising building materials and facilitating the reuse of building components. Buyle et al. (2019) believed that refurbishment could lead to the circular economy if the reuse of materials was considered. Refurbishment could contribute to the circularity of material cycles and improve cost-efficiency if proper design for reuse and good materials management were implemented. They found that metal substructure walls cost approximately 17% less than conventional walls and that the best refurbishment period was 15 years.

González et al. (2021) conducted a LCC analysis of renovation using a photovoltaic (*PV*) system. They showed that it took 25 years to payback this investment. However, when maintenance costs were included, the cumulative cash flow was negative for 45 years. Thus, final payback only occurred at year 46. Masseck et al. (2024) investigated the circular costs of using shading devices for refurbishment. They revealed up to 30% of total cost related to purchasing materials while up to 70% of cost related to assembly and disassembly. Finally, using circular economy principles could present more reliable outcomes when comparing alternatives, as it allows for the longer timespan of building usage. Considering the circular economy is recommended for future research.

## Discussion and future research

Some studies report that renovation and refurbishment are more economical than reconstructing new buildings (Ferreira et al. 2015; Power 2008; Alba-Rodríguez et al. 2017). This highlights the importance of this study to calibrate and reveal knowledge about sustainability available to the construction industry. Cost evaluation is one of the main criteria and incentives for stakeholders and plays a fundamental role in achieving sustainability goals (Dragonetti et al. 2024). Recently, there have been some attempts to simultaneously reduce the total impacts of environmental emissions and project costs (Farazmand et al. 2022). However, none of these studies document the delivery of green or fully sustainable buildings. The limitations of construction renovation or refurbishment stem from the idea of cost management. Building managers often consider the costs of renovation in the short term, while it should be considered as cost effective in the long term (Mjörnell et al. 2014). Another difficulty in calculating cost relates to the inflation rate for demolition. As demolition and deconstruction precede recycling or reuse, a different lifetime needs to be defined when predicting costs.

**Fig. 8:**
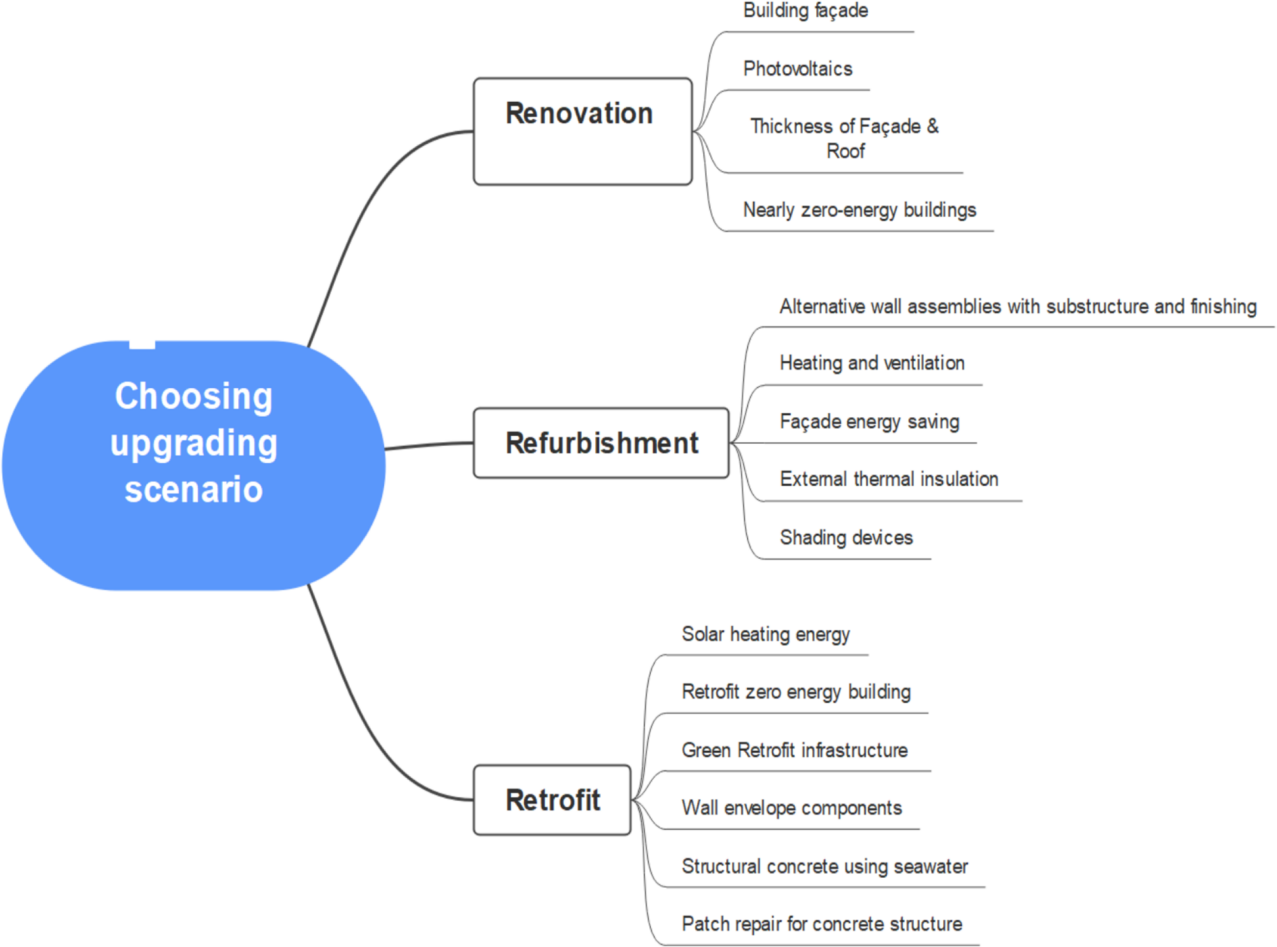
Scope of published research

One of the most critical steps in upgrading a building is choosing whether to renovate, refurbish or retrofit. The decision relies on various factors such as sustainability (Eames et al. 2014), the safety level of current structures, the building’s current load bearing capacity and the intended use (Wilkinson et al. 2014). Figure 8 illustrates building upgrade topics considered in previous research. The figure below assists in identifying areas that require evaluation in future research.

Figure 8 illustrates the areas investigated in this study. It shows that renovations have included photovoltaics or near zero-energy buildings and that these upgrades have not been considered for refurbishment. Meanwhile, solar heating, retrofitting, zero-energy buildings, green retrofit infrastructure and structural concrete using seawater have been considered as retrofit options. Thus, the key question that needs to be addressed in future research is how each upgrade scenario contributes to retrofitting, refurbishment or renovation. While there is a clear distinction between retrofitting, refurbishment and renovation, studies do not clearly explain why certain upgrades are categorized as one and not the other. Some studies use terms like retrofitting and refurbishment interchangeably (González et al. 2021; Leichter & Piccardo 2024; Niemczak et al. 2023). Among academic journals, the *Journal of Cleaner Production*, *Journal of Building Engineering* and *Journal of Building and Environment* have the highest number of publications on this topic. Geographically, research on this subject has been conducted in two countries: the USA and Italy. Moreover, most studies utilize net present value (*NPV*) as the primary method for evaluating LCC. The preference for *NPV* stems from its ability to account for opportunity costs, risk and inflation, making it a reliable tool for assessing financial sustainability (Oduyemi et al. 2016; Babashamsi et al. 2022).

The evaluation of system boundaries further reveals that most researchers assess multiple life cycle stages of buildings rather than focusing solely on specific phases such as renovation or refurbishment. However, this broad scope may reduce clarity in understanding how building upgrades impact the entire life cycle, making comparisons more challenging. Additionally, the majority of studies do not consider the end-of-life stage, including reuse, recovery and recycling of materials, despite these factors having a profound impact on total costs when evaluating renovation or refurbishment (Almusaed et al. 2024).

## Conclusion

One of the major challenges in upgrading buildings is managing costs. Several scenarios need to be considered at this stage, including renovation, repair, refurbishment and retrofitting. Each of these has a significant impact on both cost and environmental outcomes. Keyword co-occurrence analysis shows life cycle assessment (LCA) and life cycle cost (LCC) are the most frequently repeated keywords used. This indicates that most studies evaluate LCA before considering LCC. The studies were published between 2009 and 2024. The selected manuscripts highlight various attempts to incorporate life cycle cost analysis in investigations into the upgrading of buildings.

The results indicate a growing interest in life cycle cost analysis in recent years. The *Journal of Cleaner Production* and the *Journal of Building and Environment* published the highest number of articles, with five and three publications, respectively. Meanwhile, two countries—Italy and the USA—contributed the most publications in this field, each with seven journal articles. Although the articles were selected based on their focus on cost and economic evaluations, the keywords “life cycle assessment” had higher occurrences and total link strength than “life cycle cost”. This suggests that the primary focus of the selected publications was on assessing the environmental impact of renovation and refurbishment, with cost being considered a secondary objective. This could pose future challenges, as the industry’s primary concern may shift towards cost.

The result revealed that net-preset value (*NPV*) is the most applied economic indicators for analysing the economic value. Majority of studies applied renovation strategies over refurbishment and retrofit for upgrading existing structure. Previous studies believed that in order to focus on upgrading, optimization energy of building should be improved. Therefore, the researcher focused on the following strategy in upgrading existing buildings: optimizing building space, using photovoltaics, heat storage capacity or thickness of wall insulation and heat pumps.

An analysis of the selected articles has highlighted several avenues for future research. To achieve the best building upgrade solution, single-objective optimization is insufficient, and a multi-objective approach should be applied. Additionally, it is essential to consider the entire life cycle by evaluating various upgrading strategies to determine the optimal total cost. Future studies should prioritize the salvage value of materials when evaluating alternatives for upgrading existing buildings. Our analysis also indicated that most previous studies on upgrading existing buildings have neglected the financial costs associated with recycling, reusing and disposing of materials. These resources would provide greater clarity and a deeper understanding of the costs involved in upgrading buildings. Consequently, more precise cost evaluations could contribute to a higher level of sustainability.

## Data Availability

Data will be provided on reasonable request. This study is a literature review and does not involve collecting primary data. The information supporting this review was gathered from metadata and publications indexed in databases Scopus. Access to these databases is subject to their respective subscription and access policies.
